# Juvenile lupus, cytomegalovirus infection and cardiac tamponade: case report

**DOI:** 10.1590/1984-0462/2022/40/2020291

**Published:** 2021-09-01

**Authors:** Levi Coelho Maia Barros, Matheus Eugênio de Sousa Lima, Roseny Marinho Mesquita Pereira, Lia Arcanjo Alves Vasconcelos, Willenne Campelo Rabelo

**Affiliations:** a Universidade Estadual do Ceará, Fortaleza, CE, Brazil.; b Hospital Infantil Albert Sabin, Fortaleza, CE, Brazil.

**Keywords:** Lupus erythematosus, systemic, Cardiac tamponade, Cytomegalovirus infections, Lúpus eritematoso sistêmico, Tamponamento cardíaco, Infecções por citomegalovírus

## Abstract

**Objective:**

To describe a rare case of cardiac tamponade in a pediatric patient with systemic lupus erythematosus (SLE) and cytomegalovirus (CMV) infection, and to discuss the relationship between these morbidities, the diagnostic approach, and the possible treatments.

**Case description:**

A 9-year-old girl presented to the emergency department with severe dyspnea, muffled heart sounds, jugular vein distention, hemodynamic instability, and intense pallor. She had previously been followed up at the outpatient clinic for a six-month history of mild respiratory distress, polyarthritis, fever, and various cutaneous manifestations. Doppler echocardiogram revealed pericardial effusion. The patient was submitted to pericardiocentesis followed by water seal pleuropericardial drainage, with no complications. The investigation continued, with fulfillment of clinical and laboratory SLE criteria plus CMV antigenemia of 15/200,000 cells. Medications to control CMV infection and SLE were then initiated, with good clinical and laboratory response.

**Comments:**

Pediatric SLE commonly manifests in a more severe form, accounting for high morbimortality. Cardiac tamponade could be one of the first manifestations of SLE, which can also be precipitated by infectious agents, such as CMV, leading to diagnostic confusion and misleading the treatment. Changes in therapeutics must also be considered in the presence of both conditions. This study presents a juvenile SLE case aggravated by a CMV infection with the unusual manifestation of cardiac tamponade.%

## RESUMO

**Objetivo:**

Descrever e discutir um raro caso de tamponamento cardíaco na presença de Lúpus Eritematoso Sistêmico (LES) e infecção por Citomegalovírus (CMV) em um paciente pediátrico, as associações entre estas duas entidades, a abordagem diagnóstica e os possíveis tratamentos neste cenário.

**Descrição do caso:**

Paciente com nove anos de idade, sexo feminino, foi admitida no departamento de emergência com dispneia grave, hipofonese de bulhas, turgência jugular, instabilidade hemodinâmica e palidez intensa. Estava em acompanhamento ambulatorial prévio por quadro de dispneia leve, poliartrite, febre e manifestações cutâneas variadas há seis meses. Ecodopplercardiograma revelou efusão pericárdica. A paciente foi submetida à pericardiocentese seguida por drenagem pleuropericárdica em selo d’água, sem intercorrências. A investigação prosseguiu com o preenchimento de critérios clínico-laboratoriais para LES e a presença de antigenemia para CMV de 15/200.000 células. Foram iniciadas medicações para controle do LES e do CMV, com boa resposta clínico-laboratorial.

**Comentários:**

O LES pediátrico manifesta-se comumente de maneira mais grave, gerando alta morbimortalidade. Tamponamento cardíaco pode ser uma das primeiras manifestações do LES, que pode também ser precipitada por agentes infecciosos, como o CMV, levando à confusão diagnóstica e retardando o correto tratamento do paciente. Mudanças na terapêutica apropriada também devem ser consideradas na presença de ambas as condições. Com base nisso, apresentamos um caso de LES juvenil agravado por infecção por CMV e a manifestação pouco usual de tamponamento cardíaco.%

## INTRODUCTION

Childhood-onset systemic lupus erythematosus (SLE) usually exhibits more acute and severe manifestations, with a preference for the female sex and age below 10 years old.^1^ Prevalence is approximately 3.3 to 8.8 per 100,000 children,^2^ reaching up to a US$71,334 annual cost per patient depending on clinical manifestations and disease control.^3^ ^,^ ^4^ Pediatric mortality rate varies according to the severity of the disease and the quality of the provided care, being 14% along 10 years in Canada^5^ and 9% along five years in Saudi Arabia.^6^ Several studies estimate that an average of 20% of patients start having symptoms before adulthood.^7^ ^,^ ^8^

Cardiovascular manifestations are experienced by up to 54% of patients, especially as pericarditis, with no difference between ages, evolving to cardiac tamponade in around 2.5% of patients.^1^ ^,^ ^9^ Cardiac tamponade develops when pressure in the pericardium is greater than pressure in the cardiac chambers, leading to a reduction in cardiac output and, therefore, in blood pressure.^10^

Moreover, a broad range of infectious agents, such as cytomegalovirus (CMV), which are usually subclinical during childhood, gain special relevance in the context of SLE, as they can trigger flares through multiple mechanisms such as molecular mimicry, lymphocyte activation, or increased immunogenicity.^11^ In addition, either due to the very nature of this autoimmune condition or as a consequence of its treatment, the immunodeficiency state of these patients also enables reactivation and more severe manifestations of multiple infectious diseases.^12^

The aim of this study was to report a rare case in which juvenile SLE was aggravated both by CMV infection and by the unusual manifestation of cardiac tamponade. The authors provide a review on the role of CMV in cardiac involvement and as a flare trigger in SLE, in addition to discussing the management of the coexistence of these two entities, adding knowledge to the limited literature concerning this association.

## CASE REPORT

A 9-year-old girl was admitted to the emergency department (ED) presenting with respiratory distress. She has been reportedly investigated for a six-month history of less intense dyspnea and symmetrical additive polyarthritis of large and small joints (proximal interphalangeal, 2^nd^ and 3^rd^ metatarsophalangeal joints, wrists, elbows, ankles, and knees), with functional limitation and intermittent fever (38–39 °C), associated with malar rash aggravated by sun exposure, alopecia, petechiae in the trunk and neck, generalized lymphadenopathy, and weight loss not measured. Therefore, hydroxychloroquine 6.5 mg/kg/day and cefepime 100 mg/kg/day (maintained for 10 days) therapies were then started. Methylprednisolone pulse was not started by that time due to the possibility of infection.

Laboratory tests, imaging examinations, and myelogram were then performed. Laboratory results are shown in Tables 1 and 2. Chest ultrasound was performed in the ED, disclosing a 0.7cm left-side pleural effusion. It was complemented by a chest x-ray that depicted lingual atelectasis. Myelogram results revealed a hypocellular marrow, with < 1% blasts, mild dysgranulocytopoiesis, and dyserythropoiesis. The patient fulfilled Systemic Lupus International Collaborating Clinics (SLICC) 2012 criteria^13^ for SLE (presence of acute cutaneous lupus, alopecia, arthritis, serositis, hemolytic anemia, thrombocytopenia, antinuclear antibodies (ANA), anti-DNA, and antiphospholipid antibodies). Thus, prednisone 1 mg/kg and azathioprine 2 mg/kg were added to the prescription after these results.

**Table 1:** Standard exams.

Exam	Result
Hemoglobin	6.5 mg/dL
White cells	11,430/mm^3^ (Neutrophils 80% Lymphocytes 16%)
Platelets	52,900/mm^3^
Prothrombin time	1.13
Activated partial thromboplastin time	1.60
Blood urea nitrogen	7.47 mg/dL
Creatinine	0.3 mg/dL
Albumin	2.4 g/dL
Amino alanine transferase	32 U/L
Aspartate aminotransferase	90 U/L
Total Bilirrubin	0.37 mg/dL
Reactive C protein	11.40 mg/dL
Complement C3	55.8 mg/dL (reference value 50–152 mg/dL)
Complement C4	7.7 mg/dL (reference value 7–40 mg/dL)
Urinalysis	No alterations found
Direct Coombs	Positive (+)

**Table 2:** Autoimmune panel, serological exams and others.

Exam	Result
Rheumatoid Factor	nonreactive
Human leukocyte antigen B27 (HLAB27 test)	nonreactive
Antinucelar antibodies	1:640 (homogeneous nuclear pattern)
Anti-dsDNA	reactive (217 IU/mL)
Anti-Sm	nonreactive
Anti-Ro	reactive
Anti-La	nonreactive
Anti-cardiolipine IgM	reactive
Anti-cardiolipina IgG	nonreactive
Anti-beta-2-glicoprotein1	reactive
Parvovirus IgM	nonreactive
Parvovirus IgG	nonreactive
Epstein Barr Virus IgM	nonreactive
Epstein Barr Virus IgG	nonreactive
Cytomegalovirus IgM	reactive
Cytomegalovirus IgG	reactive
Tuberculin skin test	nonreactive

Three days after hospitalization, the patient progressed with a significant deterioration of general status, worsening of dyspnea, muffled heart sounds, jugular vein distension, hemodynamic instability, and intense pallor. Doppler echocardiogram was then performed, showing a large pericardial effusion with diastolic collapse of the right atrium. The patient was transfused and submitted to pericardiocentesis followed by water seal pleuropericardial drainage. Due to drain obstruction, a new approach was adopted the next day, without further complications. A new echocardiogram was performed two days later, exhibiting moderate left ventricular systolic dysfunction, an ejection fraction of 60%, and moderate tricuspid regurgitation. Furosemide 2 mg/day and spironolactone 2 mg/day therapies were then initiated. Intravenous immunoglobulin therapy (1 g/kg/day) was administered on two subsequent days due to persistent autoimmune bicytopenia. The following echocardiogram, two weeks later, showed no pathological signs.

CMV antigenemia was requested after a positive serology result, with titers of 15/200,000 cells. Ganciclovir 5 mg/kg/day therapy was started. After 10 days of treatment, antigenemia persisted (28/200,000 cells), and ganciclovir dose was therefore doubled. After eight days, another antigenemia showed titers of 1/200,000 cells. The patient continued ganciclovir 10 mg/kg/day treatment for two more weeks, evolving with good clinical and laboratory improvement, and was discharged using oral valganciclovir 20 mg/kg/day.

## DISCUSSION

SLE is an autoimmune disease characterized by multiple clinical and laboratory manifestations in consequence of a complex pathophysiology derived from immune tolerance breach, production of autoantibodies, immunodeficiency, and interactions with other pathologies, and its therapeutic arsenal has pronounced side effects.

Infections play a significant role, as they can trigger or exacerbate the disease. In addition, SLE patients are at risk for acquiring common and opportunistic infections, either due to the immunodeficiency component of the pathology (deficiencies in the complement system, polymorphisms in mannose-binding lectin, among others), or due to the immunosuppressive therapy that is the basis of SLE treatment.^14^

CMV, in immunocompetent patients, usually prompts an asymptomatic infection or a flu-like syndrome. However, when associated with SLE, it can lead to exacerbations of the disease (or even be the primary immune trigger for breaking immunological tolerance that would induce the formation of characteristic SLE autoantibodies). Furthermore, for this subset of patients, CMV infections often exhibit more severe manifestations that can be difficult to distinguish from disease flares.^15^

The prevalence of CMV infection in patients with SLE seems to be significant, although the literature on it is scarce. A study evaluating 88 lupus patients with disease complications derived from viral prime-infections concluded that CMV was responsible for approximately half of them, emphasizing its importance in patients with SLE.^16^

CMV infection in SLE may present itself as pneumonitis, myocarditis, esophagitis, hepatitis, colitis, and retinitis, as well as asthenia, polyarthritis, and erythematous papular rash, simulating disease activity.^17^ In the present report, severe cardiac involvement in the form of myocarditis and pericarditis was observed, which may be due to both lupus activity, triggered by CMV, or by the effect of CMV on the cardiac leaflets. Studies also show induction of autoantibodies of the antiphospholipid antibody syndrome in rats with SLE infected by CMV, illustrating the complexity of the interaction of these two entities.^18^

Extrapolations of treatment recommendations used for other groups of immunosuppressed patients (for instance, transplanted patients, those affected with HIV/AIDS) are common for the treatment of CMV in lupus patients, especially in the pediatric subpopulation, due to the lack of specific large studies.^19^ Among adopted strategies, it is worth mentioning the monitoring of viral antigenemia for treating the infection when it reaches a certain cutoff value. Another possible approach is the prophylactic use of antivirals (intravenous ganciclovir or oral valganciclovir) regardless of the viral load detected in immunocompromised patients. Hybrid strategies using both principles have also been used.^20^ ^,^ ^21^

A European clinical trial showed that the monitoring of viral load (via polymerase chain reaction — PCR) in pediatric patients with immunodeficiency and their respective treatment with antivirals before the onset of symptoms proved to be advantageous concerning cost reduction. However, there was no comparison between this approach and strategies that advise prophylactic use of antivirals (intravenous ganciclovir or oral valganciclovir) regardless of the viral load.^22^ In the present case, after clinical stabilization of cardiac tamponade and a positive result of pp65 antigenemia, treatment with ganciclovir was initiated, guided by its levels. Due to the persistence of the pp65 positivity, the patient was discharged using valganciclovir and will continue to be followed up as an outpatient, using the medication until antigenemia levels are negative.

Regarding cardiac tamponade, its causes are mainly divided into infectious and rheumatic diseases.^23^ Although the relationship between SLE and cardiac tamponade is epidemiologically considered rare, a retrospective study estimated its prevalence, verifying the occurrence of pericarditis in 25% of total patients, with 6.25% of them suffering from cardiac tamponade.^9^

Most studies describing this manifestation in pediatric patients consist of case reports.^24^ Maharaj et al.^23^ described 13 cases of SLE in children who presented with cardiac tamponade as their first manifestation. The authors described a 92% female patient population, and pleuritis was present in 100% of the cases. In addition, they demonstrated a strong relationship between the levels of anti-dsDNA and ANA, also evidenced by this report. Other SLE manifestations, such as nephritis, leukopenia, and consumption of complement, were described in isolated cases. The present case showed hemolytic anemia and immune thrombocytopenia associated with cardiac manifestations.

A retrospective cohort study conducted in South Africa consisting of 93 pediatric patients with SLE has found a prevalence of 47% of cardiac and vascular involvement among them, frequently severe. Twenty-four children presented with pericardial effusion, one third of them requiring intervention, with three cases of cardiac tamponade, resembling the case presentation of this report.^25^ Moreover, the prevalence of pericarditis in a Brazilian multicenter retrospective cohort study on 847 childhood-onset SLE patients was found to be 19.7%, with no significant distinction between ages.^26^

Some authors have described cases of SLE and cardiac tamponade in which all patients continued treatment with immediate pericardiocentesis guided by echocardiography, and clinical management continued with the use of high doses of corticosteroids, nonsteroidal anti-inflammatory drugs (NSAIDs), and antimalarials.^23^ ^,^ ^24^ When associated, CMV and cardiac tamponade can confuse the diagnosis and mislead the treatment. Antivirals may have pharmacologic interactions with the long-term management of cardiac complications, in addition to having its use delayed by the urgency of the clinical condition and being iatrogenically forgotten.

In view of variable clinical possibilities of lupus patients, the medical professional must consider the possibility of cardiac tamponade and its association with CMV infection, which is not only a diagnostic challenge, but also a therapeutic one, mainly due to the lack of management guidelines.
